# End-stage liver failure: filling the treatment gap at the intensive care unit

**DOI:** 10.1007/s10047-019-01133-3

**Published:** 2019-09-18

**Authors:** Robert A. F. M. Chamuleau, Ruurdtje Hoekstra

**Affiliations:** Amsterdam UMC, University of Amsterdam, Tytgat Institute for Liver and Intestinal Research, AG&M, Academic Medical Center, Meibergdreef 69-71, S1-176, 1105 BK, Amsterdam, The Netherlands

**Keywords:** Acute liver failure, Acute on chronic liver failure, Artificial liver, Liver transplantation

## Abstract

End-stage liver failure is a condition of collapsing liver function with mortality rates up to 80. Liver transplantation is the only lifesaving therapy. There is an unmet need for therapy to extend the waiting time for liver transplantation or regeneration of the native liver. Here we review the state-of-the-art of non-cell based and cell-based artificial liver support systems, cell transplantation and plasma exchange, with the first therapy relying on detoxification, while the others aim to correct also other failing liver functions and/or modulate the immune response. Meta-analyses on the effect of non-cell based systems show contradictory outcomes for different types of albumin purification devices. For bioartificial livers proof of concept has been shown in animals with liver failure. However, large clinical trials with two different systems did not show a survival benefit. Two clinical trials with plasma exchange and one with transplantation of mesenchymal stem cells showed positive outcomes on survival. Detoxification therapies lack adequacy for most patients. Correction of additional liver functions, and also modulation of the immune system hold promise for future therapy of liver failure.

## Introduction

End-stage liver failure (ESLF) is a life-threatening condition of patients with collapsing liver function, caused by massive death of liver cells. The clinical syndrome comprises bleeding risks or thrombosis, disturbed acid–base homeostasis, systemic inflammatory response, hemodynamic instability, hepatic encephalopathy (HE) with the risk of increased intracranial pressure (ICP) and multi-organ failure.

Different types of ESLF are distinguished: acute liver failure (ALF), when ESLF occurs in a person with a previous healthy liver and Acute on Chronic Liver Failure (ACLF) in a patient with an already compromised liver, mostly cirrhosis.

The incidence of ACLF in the Western World is about 70.000 patients per year and for ALF about 8000 [1]. At present, standard medical therapy consists of treating the cause of deterioration, maintaining hemodynamic stability, fluid-, acid/base- and electrolyte balance, supplying fresh frozen plasma in case of bleeding, preventing increasing ICP and, optionally hemodialysis [2].

Nevertheless, mortality rates are high, up to 80%, depending on the cause of ESLF and the number of failing organs [3]. The heterogeneity between the pathophysiology of the ESLF patients severely complicates the standardization of an effective treatment [4]. At present liver transplantation (LTX) is the only lifesaving therapy. In the EU one-year survival rates after liver LTX are 74% for ALF patients and 85% for ACLF patients (European Liver Transplant Registry 1988–2015), however, the low supply of donor livers limits the impact of LTX [5].

There is an unmet need for improving standard medical therapy to such extent that the waiting time for LTX can be prolonged and the patient enters surgery in a better condition or, ideally, that the native liver regenerates.

Different liver support strategies have been developed, including non-cell based and cell-based artificial liver supportive systems (ALSS), cell transplantation and high volume plasma exchange.

Here, we summarize the state-of-the-art of liver support strategies for ESLF and analyze remaining problems and possible solutions.

## Non-cell-based ALSS

All the non-cell-based ALSS rely on extracorporeal albumin purification, either by albumin dialysis, fractionated plasma separation, or replacement of albumin and/or adsorption techniques. These therapies aim to remove albumin-bound toxins which accumulate in the plasma. A limited number of studies has been performed on pigs with ALF caused by complete liver ischemia or overdose of acetaminophen (APAP). No ACLF models were tested. These experiments show that non-cell based albumin purification devices have the potential to improve biochemical parameters and ICP, while ADVOS and DIALIVE also improve survival time (Table 1).

**Table 1:** Large-animal studies with Non-Cell Based Artificial Liver Support devices

Supplier/expert center	Year	Device	Characteristics	Study design	Outcomes [reference]
Gambro/University Tromsø, Norway	2006	M-MARS	Modified MARS, Albumin dialysis without hemofiltration	CS in liver ischemia pigs; (*n* = 8/group)	Delayed ICP rise [53]
Gambro/University Tromsø, Norway	2007	MARS	Albumin dialysis and charcoal adsorption	CS in liver ischemia pigs; (*n* = 8/group)	Improvement of biochemistry [54]
Fresenius/Charles University, Prague, Czech Republic	2009	Prometheus	Fractionated plasma separation and adsorption	CS in liver ischemia pigs; (*n* = 14 treatment vs *n* = 8 control group)	Delayed ICP and bilirubin rise [55]
Hepa Wash GmbH/ Klinikum rechts der Isar, Technische Universität, München, Germany	2013	ADVOS	Albumin dialysis with albumin regeneration through biochemical and physical modulation	CS in liver ischemia pigs; (*n* = 6 treatment vs *n* = 5 control group)	Improvement of multi-organ failure and delayed rise of ammonia and protein-bound toxins increased short-term survival [56]
Fresenius/Charles University, Prague, Czech Republic	2014	Prometheus	Fractionated plasma separation and adsorption	CS in liver ischemia pigs; (*n* = 14 treatment vs *n* = 7 control group)	Delayed ICP rise, but no effect on hemodynamics [57]
UCL London, UK	2015	DIALIVE	Albumin exchange and endotoxin removal	CS in pigs with APAP overdose; (*n* = 9 treatment vs *n* = 7 control group)	Survival benefit [58]

Table 2 shows the results of clinical studies of non-cell based ALSS: two randomized clinical trials (RCTs), one controlled clinical trial (CCT) and one uncontrolled trial, as well as seven retrospective studies comparing two or more groups. Most of the studied treatments positively affected biochemical parameters and secondary endpoints, like HE, but whether a significant effect on the primary endpoint, i.e. improved survival rates, has been established, remains controversial [6]. A meta-analysis in 2013 of eight RCTs showed that non-cell based ALSS reduced mortality in ACLF patients (*p* < 0.018), but not in ALF patients [7]. In contrast, a meta-analysis in 2015 [8], comparing MARS treatment with standard medical therapy, showed significant effect on survival in 93 ALF patients (*p* = 0.04), and no survival effect in 453 ACLF patients. Subsequent clinical studies continued to produce contradictory results with albumin dialysis systems. Gerth et al. [9], in a retrospective study of 101 ACLF patients, confirmed improved short-term mortality in ACLF by MARS, but the same group failed to improve 28-day mortality in ALF [10]. These apparent contradictory results need further clarification. It is most likely that only specific subgroups of ESLF patients, i.e. those with less severe liver failure, may profit from non-cell based ALSS. A combination with plasma exchange (PE) seems to improve the impact of non-cell based ALSS therapy; controlled studies on non-cell based ALSS showed predominantly improved survival in those combination therapies (2 out of 2 studies [11, 12]), while stand-alone non-cell based ALSS therapies predominantly failed to provide any survival benefit (negative studies: [10, 13–15]) with the exception of two positive studies [9, 16].

**Table 2:** Clinical studies with non-cell based artificial liver support systems

Supplier/expert center	Year	Device	Characteristics	Study design	Outcomes [reference]
Zhejiang University, Hangzhou, China	2005	ALSS	Plasma exchange, charcoal adsorption, bilirubin adsorption	CCT in ACLF patients (*n* = 338 treatment *vsn* = 312 controls)	Improved 30-day survival and biochemistry [11]
Gambro-Baxter/Jena University, Germany	2009	SPAD and/or MARS	SPAD: Single –Pass Albumin Dialysis MARS: Albumin dialysis and charcoal adsorption	Retrospective comparison in ALF/ACLF patients with MARS (*n* = 33), SPAD (*n* = 12) or MARS & SPAD combined (*n* = 12)	Biochemical improvement from baseline [59]
Fresenius/University Duisburg-Essen, Germany	2012	Prometheus	Fractionated plasma separation and adsorption	RCT in ACLF patients (*n* = 77 treatment *vsn* = 68 controls)	Biochemical improvement, no survival benefit [13]
Gambro-Baxter/Hospital General, Madrid, Spain	2013	MARS	Albumin dialysis and charcoal adsorption	RCT in ACLF patients (RELIEF) (*n* = 95 treatment *vs* 94 controls)	No survival benefit [14]
Gambro-Baxter/University of Muenster, Germany	2017	MARS	Albumin dialysis and charcoal adsorption	Study 1. Retrospective comparison of ACLF patients (*n* = 47 treatment *vsn* = 54 controls) Study 2 CCT in ALF/graft dysfunction patients (*n* = 32 treatment *vsn* = 41 controls)	Study 1. Biochemical and clinical improvement and improved short-term survival [9] Study 2 No survival benefit [10]
Hepa Wash GmbH/Klinikum rechts der Isar, Technische Universität, München, Germany	2017	ADVOS	Albumin dialysis with albumin regeneration through biochemical and physical modulation	Uncontrolled study in ACLF patients (*n* = 9)	Biochemical improvement from baseline [60]
Gambro-Baxter/University Maryland, Baltimore, USA	2017	MARS	Albumin dialysis and charcoal adsorption	Retrospective, uncontrolled study in ALF patients (*n* = 27)	Improvement of grade of HE [61]
Nephrology Dept, Dali University, Chuxiong, China	2018	PD or PE	Peritoneal Dialysis or plasma exchange	Retrospective comparison in ALF and ACLF patients, PD (*n* = 22), PE (*n* = 28) *vs* controls (*n* = 12)	PD improved survival and biochemistry [16]
Capital Medical University, Beijing, China	2018	DPMAS and PE	Double plasma molecular adsorption and plasma exchange	Retrospective comparison of HBV-ACLF patients, DPMAS + PE (*n* = 54), *vs* PE (*n* = 77)	Improved bilirubin and 28-day survival rate [12]
Fresenius/University of Lodz, Poland	2019	Prometheus SPAD	Coupled plasma filtration and adsorption or SPAD	Retrospective comparison ESLF patients (*n* = 48 treatment *vsn* = 53 controls)	No difference in 3-month mortality [15]
Graduate School of Medicine, Chiba, Japan	2019	CHDF	Continuous hemodiafiltration and plasma exchange	Retrospective comparison of fulminant hepatitis patients (*n* = 47) *vs* controls (*n* = 45)	Improvement of grade of HE [62]

In summary, different albumin purification devices unequivocally reduce elevated plasma bilirubin, and haemodialysis reduces plasma ammonia levels. Consequently, HE grade might be improved. This reduction of HE, may, in combination with removal of albumin-bound toxins ameliorate ESLF. According to the meta-analyses [7, 8] in a minority of cases, non-cell based ALSS improved survival time, but it is unlikely that stand-alone non-cell based ALSS will prevent liver transplantation, as normalization of coagulation, electrolyte balance, body homeostasis and cardiovascular stability requires a more complete restoration of failing liver function. Nevertheless, new systems, like DIALIVE and ADVOS, which show short-term survival benefit in animals with ALF, are under investigation, but we have to wait for their clinical benefit on survival.

## Cell-based ALSS

### Bioartificial liver (BAL) devices

A BAL is an extracorporeal device containing liver cells to be connected temporarily to the patient’s circulation compensating the failing detoxification, synthetic and homeostatic function of the diseased liver. An optimal BAL device promotes liver cell differentiation by allowing medium perfusion, 3-dimensional growth and supplying oxygen and contains sufficient liver cell mass (at least 20% of the native liver, approximating 15.10^9^ or 150 g hepatocytes) that provides stable support of multiple liver-specific functions [17]. Therefore, a BAL has more potency to be effective than non-cell ALSS. Besides highly functional, the ideal biocomponent of BALs should be of human origin, safe and cost effective.

Many BAL-devices have been studied in experimental models of large animals with ALF (Table 3); only few have reached clinical application (Table 4).

**Table 3:** BAL devices in preclinical development in large animal models

Supplier/expert center	Year	Device	Characteristics	Study design	Outcomes [references]
Vital Therapies/Baylor College of Medicine, USA	1994	ELAD	Hollow fiber bioreactor with C3A cells (200 g)	Uncontrolled study in three dogs with total hepatectomy and five dogs with overdose APAP	Safety shown [63]
HepArt Medical Devices/University of Amsterdam, the Netherlands	1999/2002	AMC-BAL	Perfused scaffold bioreactor with primary porcine hepatocytes (14 billion in first study and 10.7 billion in second study) in 3D	Two CS’s in liver ischemia pigs (c: *n* = 10, eB: *n* = 5, cB: *n* = 8) and anhepatic pigs (c, eB & cB: *n* = 5)	Improved survival time and biochemistry [64, 65]
Los Angeles School of Medicine, USA	2001	HepatAssist	Cryopreserved porcine hepatocytes (10 billion) and charcoal adsorption	CS in liver ischemia pigs (c, eB & cB: *n* = 5)	Delayed ICP rise, improved survival time [66]
Hong Kong Ltd Co/PLA General Hospital, Beijing, China	2001	TECA-1 BALSS	Hollow fiber, freshly isolated porcine hepatocytes (10 billion)	CS in dogs partial hepatectomy (cB: *n* = 10, eB: *n* = 4)	Improved survival time and biochemistry [67]
Jichi Medical School, Japan	2005	Circulatory flow bioreactor	Cytochrome P450 3A4 and Glutamine Synthetase overexpressing HepG2 cells (100 million)	CS in dogs with liver ischemia and diazepam overdose (c: *n* = 3, eB: *n* = 7, cB: *n* = 8)	Improved survival time [68]
National Research Institute Tokyo, Japan	2006	Circulatory flow bioreactor	Glutamine Synthetase overexpressing HepG2 cells (3.5–4.1 billion)	CS in liver ischemia pigs (c: *n* = 8, eB: *n* = 9, cB: *n* = 8)	Improved survival time and biochemistry, delayed ICP rise [69]
Thomas E. Starzl TX Institute Pittsburgh, USA	2007	Excorp Medical BLSS	Hollow fiber, 70 g primary porcine hepatocytes, blood perfusion	CS in dogs with D-GALN overdose (c: *n* = 6, cB: *n* = 9, no eB)	Reduced metabolic acidosis [70]
Southern Medical University, Guangzhou, China	2014	HBALSS	Human cell bank liver cells (HL-7702) (4 billion) in microcarriers in perfusion bioreactor	CS in cynomolgus monkeys with D-GALN overdose (c: *n* = 5, cB: *n* = 10, no Eb)	Improved survival time and biochemistry [71]
Mayo Clinics, USA	2015	SR-BAL	Porcine hepatocyte spheroids (59–228 g) plus plasma filtration	CS in pigs with D-GALN overdose (c, eB & cB: *n* = 6)	Improved survival and biochemistry, delayed ICP rise [72]
University of Compiègne, France	2015	Suppliver	Fluidized bed bioreactor with detoxification columns, alginate encapsulated C3A cells (15% liver mass)	Uncontrolled study in two healthy sheep	Safety shown [73]
University Shanghai, China	2016	hiHep-BAL	Radial flow bioreactor with 65 flat layers (2D); hiHep cells (transdifferentiated human fibroblasts) (3 billion)	CS in eight mini-pigs with D-GALN overdose (c: *n* = 6, eB: *n* = 6, cB: *n* = 8)	Prolonged survival and improved biochemistry [74]
Hangzhou University, China	2016	FBBAL	Fluidized bed, alginate encapsulated primary porcine hepatocytes (5 billion)	CS in pigs with D-GALN overdose (c, eB & cB: *n* = 7)	Change in serum metabolome and prolonged survival time [75]
University College London, UK	2017	UCLBAL	Fluidized bed-based bioreactor with alginate beads of human HepG2 cells (70 billion)	CS in liver ischemia pigs (eB: *n* = 16, cB: *n* = 13)	Biochemical improvement and delayed ICP rise [34]
Samsung Medical Center, Korea	2017	LifeLiver	Alginate encapsulated primary porcine hepatocytes (20 billion)	CS in liver Ischemia pigs(c, eB & cB: *n* = 5)	Prolonged survival time, improved biochemistry and delayed ICP rise [76]
Mayo Clinics, USA/ Sichuan University, China	2018	SRBAL	Porcine hepatocyte-human umbilical vein endothelial cell organoids (25.6 ± 4.1 billion cells) plus plasma filtration	CS in monkeys with α–amanitin and lipopolysaccharide intoxication (c, eB, cB: *n* = 6)	Improved survival time and biochemistry [77]
Mayo Clinics USA	2019	SRBAL	Primary porcine hepatocytes spheroids (200 g) plus plasma filtration	CS in post-hepatectomy pigs (c, eB, cB: *n* = 6)	Improved survival time, neuroprotective benefit, improved biochemistry and accelerated liver regeneration [78]

**Table 4:** Clinically applied BAL devices

Supplier/expert center	Year	Device	Characteristics	Study design	Outcomes [references]
Beijing and Nanjing Universities, China	2001	TECA-HALSS/HBAL	Hollow fiber hybrid BAL, charcoal adsorption and primary porcine hepatocytes (10–20 billion)	Uncontrolled study, six ALF and three ACLF patients	Safety, improved grade of HE [79]
Thomas E. Starzl TX Institute Pittsburgh, USA	2002	BLSS	Hollow fiber, primary porcine hepatocytes (70–100 g)	Uncontrolled study, one ALF patient	Safety shown [80]
Charité, Berlin, Germany	2003	Cell Module Bioreactor	Modular Extracorporeal Liver Support; primary porcine hepatocytes (1.8–4.4 billion)	Uncontrolled study, eight ALF patients	Successfully bridged to transplantation [23]
Cedars Sinai Medical Center, Los Angeles, USA	2004	HepatAssist	Cryopreserved porcine hepatocytes (7 billion) and charcoal adsorption	RCT, ALF patients treated (*n* = 85 treatment *vsn* = 86 control group)	No survival benefit by intent-to–treat [18]
HepArt Medical devices B.V./ University of Amsterdam, the Netherlands	2005	AMC-BAL	Perfused scaffold; primary porcine hepatocytes (7–15 billion) in 3D	Uncontrolled study, twelve ALF patients	11/12 successfully bridged to transplantation and 1/12 spontaneous recovery [81]
Vital Therapies/ University of Minnesota, Minneapolis, USA	2018	ELAD	Hollow fiber, 440 g C3A cells	RCT, severe alcoholic hepatitis patients (*n* = 96 treatment *vsn* = 107 control group)	No survival benefit by intent-to-treat [20]

### Preclinical BAL studies

The applied large animal models relied on ALF caused by complete liver ischemia, total hepatectomy, overdose of drugs, such as D-galactosamine (D-GALN) and APAP, or intoxication with α-amanitin and lipopolysaccharide. Most controlled studies (CSs) included three groups; control with no connection to the system (c), empty-BAL (eB) and cell-filled-BAL (cB). Studies that did not include the eB group are less informative, as dilution of plasma or blood by pre-filling the extracorporeal circuit may lead to attenuation of ESLF. Most CSs in animals (13 out of 15) showed a beneficial effect on survival and biochemical markers. One study even reported that BAL treatment accelerated liver regeneration (56). So far, BAL systems have not been studied in animal models of ACLF.

### Clinical BAL studies

Four different BAL systems based on freshly isolated porcine liver cells (BLSS, MELS, AMC-BAL and TECA-BALSS/HBAL) were tested in phase I/IIa studies and one, the HepatAssist, with cryopreserved porcine liver cells, in an RCT. All studies showed the safety of the system. However, the RCT with the HepatAssist device could not establish survival benefit in the whole patient population, analysed by intention to treat. Unexplained was an increased survival in the subpopulation of fulminant/subfulminant hepatic failure patients [18]. In the uncontrolled study of the AMC-BAL plasma levels of total bilirubin and ammonia decreased by 35 and 45%, respectively, and was associated with improved neurological state and stabilization of hemodynamics [19].

The ELAD system, based on the human liver cell line C3A, has been extensively tested in clinical trials with the largest ones being NCT01471028, an open-label RCT in 203 patients with severe alcoholic hepatitis [20], and very recently VTL-308 in 151 less severe and relatively young alcoholic hepatitis patients (age > 18 and < 50 years, MELD (Model for End-Stage Liver Disease) score < 30). Both RCTs showed temporary improvement of some biochemical parameters in ELAD-treated groups *vs* control groups, but no significant benefit on survival. By intent to treat analysis, ELAD did not meet its primary endpoint, overall survival (https://vitaltherapies.com/research-clinical-trials-development/elad-rostock-9-29-18/).

### Concluding remarks clinically applied cell-based ALSS

Most BAL systems showed safety and efficacy in animal models of ESLF, indicating that supply of a broad spectrum of liver functions has a high potential to support ESLF patients. However, a clinical breakthrough has not yet been obtained, due to different factors. In Europe progress has been delayed due to the European moratorium on xenotransplantation [21]. The major concerns around BAL applications with xenogeneic liver cells included immunological responses and risks associated with zoonotic infections. So far, transmission of porcine endogenous retroviruses to patients treated with BAL containing porcine cells has not been observed [22, 23]. Consequently, outside Europe researchers continue developing BALs based on porcine hepatocytes; the SR-BAL and LifeLiver systems are pre-clinically under investigation (Table 3).

As an alternative for primary porcine hepatocytes, human liver cell lines have been most extensively applied as biocomponent for BALs. Their human origin, high reproducibility and relatively cheap propagation make them more suitable for BAL application. However, the cell lines most frequently used until now, C3A and HepG2, display a limited spectrum of liver functions and even produce lactate and ammonia, instead of eliminating it [17]. This may be a reason for the disappointing outcomes of the clinical ELAD trials. Another cell source comprises original or induced stem cells, which, however, display a limited functional spectrum as well [17, 24]. To date, the most functional human liver cell line is the HepaRG cell line [25], which further differentiates by culturing in the AMC-BAL system [26]. Comparison of the transcriptomes of different proliferative sources of human liver cells showed that HepaRG cells most closely resembled primary human hepatocytes [24, 27]. Further improvement of existing cell systems may be achieved by (conditional) ectopic expression of limiting regulatory or structural genes and by application of cell culture systems promoting maturation, e.g. by delivering high oxygen levels, 3D configuration and perfusion [28].

The development of BAL technology also faces a logistical challenge, as cells need to be provided with preservation of high functionality at the bedside. Cryopreservation of primary hepatocytes, however, may further aggravate the damage already induced by isolation [29], leading to cell death or dedifferentiation. This may be the reason for the disappointing results of the HepatAssist RCT [18]. Cryopreservation is substantially less damaging for proliferative cells than mature cells. In addition, liver cells rapidly deteriorate in the presence of human plasma, which limits the therapeutic window of the BAL [30, 31]. This negative effect can be reversed by an intermittent phase of recirculating culture medium through the bioreactor [32]. Another challenge that extracorporeal BALs face is the provision of in vivo like bile secretion [33]. A hybrid BAL containing a sufficient amount of well-functioning liver cells combined with integrated haemodialysis or albumin purification might be the preferred option to improve the patient’s condition to create time for native liver regeneration or liver transplantation. It is evident that for clinical application, the cell- and BAL-cultures need to be produced according to Good Manufacturing Practice [34].

Together, these challenges render a BAL system a complex product, and the costs of a BAL treatment will be substantial. This is actually a general problem for advanced therapies, based on cell or tissue-engineered products.

## Other therapies

### High volume plasma exchange

By plasma exchange combined with hemoperfusion or continuous hemodiafiltration overabundant toxic substances will be removed and reduced liver-specific products will be replenished. To our knowledge, no preclinical survival studies in large animals have been performed. The first large prospective CS in HBV-associated ACLF patients (MELD score 28–29) showed higher short- and long-term survival in the treatment group (*n* = 104) *vs* the control group (*n* = 130) [35].

In 2016 Larsen et al. [36] showed in a RCT that high volume plasma exchange (HVPE), with 8–12 L daily volume exchange, improved survival in patients with ALF (mainly APAP overdosed) by correcting the hemodynamics and biochemistry, e.g. the ammonia levels, and by modulating the innate and adaptive immune responses to the necrotic liver [37]. These two studies indicate a beneficial effect of PE or HVPE on ESLF patients. Further studies are needed to consolidate these results.

### Hepatocyte or stem cell transplantation

Based on several successful survival studies in large animals [38–40], cell transplantation has been studied as an alternative way of filling the treatment gap at the ICU for ESLF patients [41].

Initially, hepatocytes were the cell source of choice for cell transplantation of ESLF patients. Five patients with ALF underwent intrasplenic or/and intrahepatic hepatocyte transplantation. All patients showed temporary clinical and biochemical improvement but eventually died [42]. A severe complication is that for treatment of ESLF large amounts of successfully engrafting and safe hepatocytes are needed. Therefore, hepatocyte transplantation is a more promising strategy for correcting liver-based metabolic deficiencies [43], requiring lower amounts of engrafted cells, and is less suitable for ESLF therapy.

Besides hepatocytes, mesenchymal stem cells (MSCs) have also been applied in cell transplantations in preclinical survival studies in large animals [44–49]. These cells do not directly support liver functions, but rather produce paracrine factors (e.g. cytokines, chemokines, and growth factors) with immunomodulatory and liver regeneration promoting effects [50].

A controlled study in HBV-associated ACLF patients compared the outcomes of four groups: controls (*n* = 30): PE (*n* = 30), umbilical cord-MSC transplantation (*n* = 30) and UC-MSC + PE (*n* = 20) [51]. It was concluded that MSC transplantation combined with PE treatment was safe, but could not significantly improve the short-term prognosis of HBV-ACLF patients compared to the single treatment.

An RCT on HBV-related ACLF patients showed that four weekly infusions with 100,000–1 million MSCs improved the MELD score and bilirubin levels, decreased the incidence of severe infections and increased the 24-week survival [52]. These studies indicate promising results. However, further studies are needed to show the benefit of MSC transplantation in all categories of ESLF patients.

## Concluding remarks

Interesting developments are ongoing in the field of liver support for ESLF patients. From the first non-cell-based ALSS studies we learned that detoxification modalities may temporarily yield improvement of biochemistry and grade of HE, but the supply of plasma factors, control of homeostasis, and/or modulation of the immune system are needed to effectively support ESLF patients.

BAL systems were able to improve survival in experimental animal models of ALF, but due to practical and financial problems and usage of cells with low functionality, the clinical development remains behind. Other systems as PE and MSC transplantation, both modulating the immune responses, with PE also supplying detoxification and plasma proteins, hold promise for the future as well, as survival benefit has been shown in clinical trials.

The question rises which therapy should be used in which patients and at which stage of the disease? Table 5 compares advantages and disadvantages of different treatment modalities for ESLF patients. Apart from providing survival benefit also other factors are relevant for decision making, including the complexity, risks and costs of the procedure, and the status of the patient. For the future, we need improved metabolic and immunologic monitoring of ESLF patients in combination with detailed measurement of the effect of the different therapies. Subsequently, therapies can be selected on the basis of informative biomarkers. This will progress the care of ESLF patients towards a more patient-tailored approach, optionally by combining different treatment modalities at different stages of the disease.

**Table 5:**
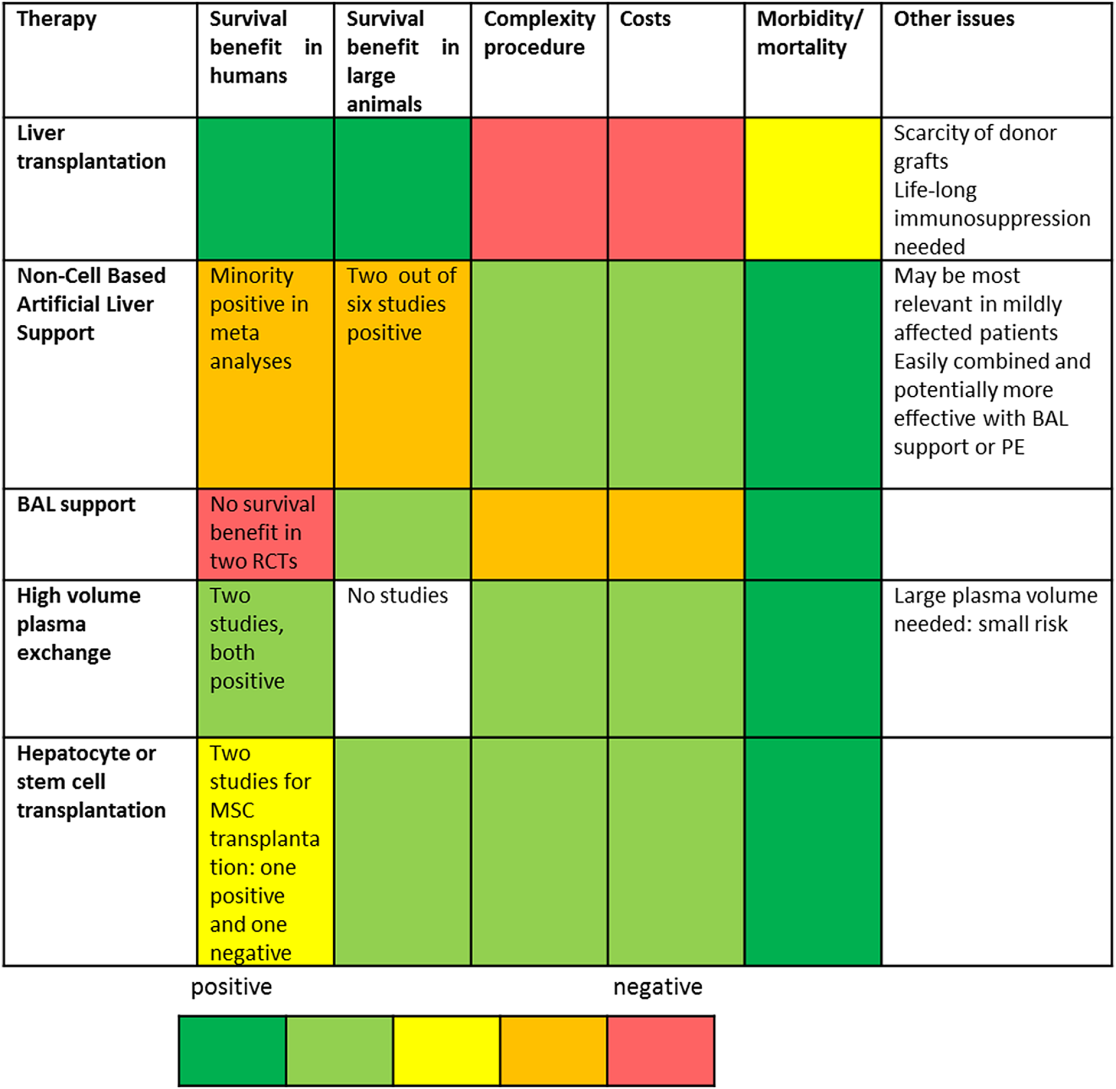
Comparison of different therapies for ESLF patients
